# Assessment of Healthy and Harmful Maillard Reaction Products in a Novel Coffee Cascara Beverage: Melanoidins and Acrylamide

**DOI:** 10.3390/foods9050620

**Published:** 2020-05-12

**Authors:** Amaia Iriondo-DeHond, Ana Sofía Elizondo, Maite Iriondo-DeHond, Maria Belén Ríos, Romina Mufari, Jose A. Mendiola, Elena Ibañez, Maria Dolores del Castillo

**Affiliations:** 1 Instituto de Investigación en Ciencias de la Alimentación (CIAL) (CSIC-UAM), Calle Nicolás Cabrera, 9, 28049 Madrid, Spain; amaia.iriondo@csic.es (A.I.-D.); anasofi.eliz@gmail.com (A.S.E.); rios.mbelen2019@gmail.com (M.B.R.); romi_mufari@hotmail.com (R.M.); j.mendiola@csic.es (J.A.M.); elena.ibanez@csic.es (E.I.); 2 Instituto Madrileño de Investigación y Desarrollo Rural, Agrario y Alimentario (IMIDRA), N-II km 38, 28800 Alcalá de Henares, Spain; maite.iriondo@madrid.org; 3 Instituto de Ciencia y Tecnologia de los Alimentos (ICTA), Av. Velez Sarsfield 1611, Cordoba 5016, Argentina

**Keywords:** acrylamide, coffee cascara, food safety, instant beverage, Maillard reaction, melanoidins

## Abstract

Our research aimed to evaluate the formation of Maillard reaction products in sun-dried coffee cascara and their impact on the safety and health promoting properties of a novel beverage called “Instant Cascara” (IC) derived from this coffee by-product. Maillard reaction products in sun-dried coffee cascara have never been reported. “Instant Cascara” (IC) extract was obtained by aqueous extraction and freeze-drying. Proteins, amino acids, lipids, fatty acid profile, sugars, fiber, minerals, and vitamins were analyzed for its nutritional characterization. Acrylamide and caffeine were used as chemical indicators of safety. Colored compounds, also called melanoidins, their stability under 40 °C and in light, and their in vitro antioxidant capacity were also studied. A safe instant beverage with antioxidant properties was obtained to which the following nutritional claims can be assigned: “low fat”, “low sugar” “high fiber” and “source of potassium, magnesium and vitamin C”. For the first time, cascara beverage color was attributed to the presence of antioxidant melanoidins (>10 kDa). IC is a potential sustainable alternative for instant coffee, with low caffeine and acrylamide levels and a healthy composition of nutrients and antioxidants.

## 1. Introduction

Maillard Reaction Products (MRPs) are the result of a chemical reaction between amino acids and reducing sugars when foods are being processed at high temperatures [1]. This reaction enhances flavor and color, and MRP have been associated with both positive and negative health effects [2]. In the case of coffee, the main MRPs produced during coffee roasting are melanoidins. The main sources of dietary melanoidins in Western diets are coffee and bakery products [3,4]. The average melanoidin content in medium roasted coffee is 7.2 g per 100 g. On the other hand, the amount of melanoidins in bread crusts ranges from 14 g to 30 g per 100 g of bread crust [3]. Different health-promoting properties such as antioxidant, antimicrobial, anti-inflammatory and antihypertensive activity have been assessed in these molecules [5]. Besides melanoidins, another MRP found in coffee beverages is acrylamide. This compound is considered a contaminant and classified by the International Agency of Research on Cancer (IARC) as a potential carcinogenic (class 2A) [6]. The European Food Safety Authority (EFSA) has stated that coffee and its substitutes can contribute up to 40% of the dietary exposure to acrylamide for the adult population. The European Commission (EC) established several recommendations for monitoring acrylamide levels in food, recommending 450 μg/kg for roasted coffee and 900 μg/kg for instant coffee [7]. Besides coffee, the main sources of human dietary exposure to acrylamide are fried potatoes (~272–570 μg/kg), bakery products (~75–1044 μg/kg) and breakfast cereals (~149 μg/kg) [7,8].

Given the great demand for coffee worldwide, an important number of by-products are generated during its processing [9]. Coffee cascara represents the main by-product of the coffee industry and its revalorization has gained interest over the last decade. After de-pulping, this by-product is normally dehydrated in the sun for 21 days to reduce its moisture to 10%. Cascara contains the substrates, such as amino acids, proteins and carbohydrates, needed for the Maillard reaction (MR) [10]. During the dehydration process of cascara, the MR may occur and healthy (melanoidins) and harmful (acrylamide) MRP may be generated [11,12]. The occurrence of the MR during the dehydration of figs, dates and raisins under similar conditions to those employed in cascara processing has been previously described [11].

Recent studies consider cascara as a potential source of phenolic compounds with antioxidant properties and the potential to improve human health [13,14]. For this reason, the use of this by-product to elaborate a novel antioxidant beverage supposes several advantages: promoting the sustainability of the coffee industry, avoiding the waste of new by-products by using the insoluble residue that results from the extraction to obtain cascara flour [10], and creating an added value, sustainable and healthy drink [15]. Since cascara is considered a novel food according to the European Commission [16], more safety and toxicity studies are needed for its further approval [17].

An aqueous extraction of coffee cascara was proposed to obtain a soluble powder also called hereby as ‘Instant Cascara’ (IC). This research seeks to obtain a product that offers a healthier nutritional profile than the powdered soft drinks commercially available, which stand out mainly for the excessive presence of sugar [18]. A very recently published critical review showed that the consumption of soft drinks has increased dramatically over the past years, being mostly consumed by children and teenagers [19]. Excessive intake of soft drinks with high sugar and acid leads to dental caries and erosion, overweight, obesity and increased risk of type 2 diabetes [19]. Although consumers are aware of the impact of the consumption of these beverages on human health, it is still necessary to educate the population about the harmful effects of these drinks and also to develop healthier alternatives. Therefore, the aim of this research was to develop a novel safe antioxidant “Instant Cascara” beverage (IC), contributing to the sustainability of the coffee sector and offering healthier products to the general population satisfying their nutritional demands. To achieve the goal, the formation of Maillard reaction products in sun-dried coffee cascara and their impact on the safety and health promoting properties of a novel beverage called “Instant Cascara” (IC) derived from this coffee by-product was assessed. Maillard reaction products in sun-dried coffee cascara have never been reported before.

## 2. Materials and Methods

### 2.1. Food Samples

#### 2.1.1. Raw Cascara

SUPRACAFÉ S.A. (Móstoles, Madrid, Spain) provided coffee cascara (CA) from Arabica species and Tabi variety from Colombia. Coffee cascara was obtained in the processing of the coffee berry, dried for 21 days in the sun, and subjected to a sanitation process involving the use of a carbon dioxide atmosphere (Martin Bauer, MABA-PEX process).

#### 2.1.2. Homemade Instant Cascara

Powdered aqueous extract from coffee cascara (IC) was obtained as described in the patent WO2013004873A1 [20], which consisted of an aqueous extraction of 50 g/L at 100 °C for 10 min. Sample was filtered (250 μm) and freeze-dried. IC extraction yield was 20%.

Two beverages were formulated from IC at 4 mg/mL and 10 mg/mL. Concentrations were chosen based on commercial instant coffee drinks. Elaboration consisted of diluting each IC dose with water at room temperature.

#### 2.1.3. Commercial Cascara Infusion (Tabifruit)

IC drinks (4 and 10 mg/mL) were compared with a commercial infusion of coffee cascara (Tabifruit, Supracafé S.A., Madrid, Spain). This was elaborated according to the procedure indicated for the product, leaving the infusion bag (3 g) in 250 mL of water at 100 °C for 4 min, creating a final concentration of cascara of 12 mg/mL.

### 2.2. Nutritional Characterization

#### 2.2.1. Protein and Amino Acid Profile

Protein content in cascara (CA) and instant cascara (IC) was obtained through Kjeldahl mineralization carried out by the Bioanalytical Techniques Unit at the Instituto de Investigación en Ciencias de la Alimentación (CIAL, UAM-CSIC, Madrid, Spain) and quantification was performed by colorimetric analysis of nitrogen (AOAC-32.1.22,920.87). A conversion factor of 5.6 was used to calculate protein content. Analysis was carried out in duplicate and results were expressed as percentage of dry matter.

Free and total amino acid quantification was carried out by the Servicio de Química de Proteínas of the Centro de Investigaciones Biológicas (CIB, CSIC, Madrid, Spain). For total amino acid quantification, samples were hydrolyzed in an acid medium for later analysis by high performance liquid chromatography (HPLC) with post-column derivatization, using nihydrin. Analyses were carried out in duplicate and results were expressed as mg/g.

Both determinations were performed as previously described [21].

#### 2.2.2. Lipids and Fatty Acid Profile

Lipid quantification in CA and IC was performed by Soxhlet extraction as described in AOAC Official Method 945.16. using petroleum ether. Results were obtained by weighing the dry product containing the lipid fraction and were expressed as % dry matter.

Fatty acid profile was determined by gas chromatography according to ISO 12966-2:2017; using a flame ionization detector (Agilent 7820A GC system, Agilent Technologies, Inc., Santa Clara, CA, USA) [22]. Analysis was carried out by the Analysis Services Unit facilities of the Institute of Food Science, Technology and Nutrition (ICTAN, CSIC, Madrid, Spain). Analysis was carried out in duplicate and results were expressed as grams per 100 g of sample.

Both determinations were performed as previously described [23].

#### 2.2.3. Dietary Fiber

Insoluble (IDF), soluble (SDF) and total (TDF) dietary fiber were determined in CA and IC using the Megazyme Total Dietary Fiber Kit (Megazyme, Wicklow, Ireland), an enzymatic-gravimetric assay based on the AOAC-991.43 and AACC-32.07.01 method. Analysis was carried out in duplicate. Results were expressed as weight percentage (%).

#### 2.2.4. Sugars, Minerals and Vitamin C

Determination of sugars, minerals and ascorbic acid in CA and IC was carried out by the Analysis Services Unit facilities of the Institute of Food Science, Technology and Nutrition (ICTAN, CSIC, Madrid, Spain). All determinations were performed in duplicate as previously described [24]. Results were expressed as g/100 g for simple sugars and mg/100 g for minerals and ascorbic acid.

#### 2.2.5. Total Carbohydrates

Carbohydrate content in liquid IC beverages (4 mg/mL and 10 mg/mL) was determined as described by Masuko et al. (2005), using the phenol-sulfuric method [25]. The experiment was initiated with the preparation of the reagents, phenol at 5% (Sigma-Aldrich, St. Louis, MO, USA) and sulfuric acid at 98% (Sigma-Aldrich, St. Louis, MO, USA). Glucose (Sigma-Aldrich, St. Louis, MO, USA) calibration curves was prepared (0.1–0.5 mg/mL), and in 2 mL glass vials, 100 μL of sample or standard, 300 μL of sulfuric acid and 90 μL of phenol or H_2_0 for the sample blanks were added. Vials were incubated at 90 °C for 5 min, followed by a water bath at room temperature for 5 min. Then, 100 μL of each sample was transferred to a 96-well plate and absorbance was measured at 490 nm in a UV-Visible spectrophotometer (BioTek Instruments, Winooski, VT, USA).

#### 2.2.6. Glucose

Free glucose content was determined in liquid IC beverages (4 mg/mL and 10 mg/mL) using the Glucose TR Kit (SpinReact, Girona, Spain) following manufacturer’s instructions. Results were expressed as mg glucose/ mL of beverage.

### 2.3. Maillard Reaction Products (MRP)

#### 2.3.1. Acrylamide

For certifying the safety of the novel instant beverage, acrylamide content was analyzed in the IC powder and in liquid samples. A sample preparation procedure according to the standard UNE-EN 16618:2015 [26] with some modifications was used. A total of 1 g of sample was weighed into a centrifuge tube (20 mL of water in the case of IC powder were added) and was spiked with 100 ng/g of ^13^C_3_-labelled acrylamide (Sigma-Aldrich, St. Louis, MO, USA), to determine the percentage recovery of the method at this stage. Then, it was quantitatively transferred for the SPE clean-up in C18 (Isolute, 500 mg/ 6 mL, Biotage, Uppsala, Sweden) cartridge preconditioned using a multi-stage vacuum system (Agilent Technologies; Palo Alto, CA, USA). The eluate was collected and submitted to another step of clean up with the ENV+ (Isolute, 500 mg/ 6 mL, Biotage, Uppsala, Sweden) preconditioned cartridge. The cartridge was rinsed with two fractions of 2 mL of water, followed by the elution of acrylamide with 2 mL of methanol 60% in water. The final extract was concentrated by removing the extraction solvent in an oven at 35 °C to an approximate volume of 500 µL and stored in vials for the quantitation by LC-MS/MS.

The LC-MS/MS analysis was performed according to methodology described by Mastovska and Lehotay [27], using an Agilent 1290 Infinity II system, interfaced to an Agilent 6470 triple quadrupole mass spectrometer (Agilent Technologies; Palo Alto, CA, USA). Sample injection volume was 10 µL, and an Agilent Infinity Lab Poroshell 120 EC-C18 column (100 × 4.6 mm; 2.7 µm particle size) kept at 30 °C was employed for the LC separation. The mobile phase was 99.5:0.5 (*v*/*v*) water-MeOH, with 0.1% formic acid, at a flow rate of 400 µL/min for 8 min for elution of acrylamide (retention time 4.02 min) and then 0.1% formic acid in MeCN-MeOH (50:50, *v*/*v*) for the post-analysis wash (at 400 µL/min for 6 min) followed by equilibration to initial conditions. The MS determination was performed in ESI positive mode. Monitoring transitions *m*/*z* 72 > 55, 72 > 54, 72 > 44, and 72 > 27 were recorded for acrylamide, whereas the transitions *m*/*z* 75 > 58 were used for the ^13^C_3_-labelled acrylamide. Results were expressed as μg/kg of sample.

#### 2.3.2. Melanoidins

The content of melanoidins in liquid IC beverages was analyzed spectrophotometrically. Light-absorption measurement of samples at 420 nm was performed using a microplate reader (BioTek Epoch 2 Microplate Spectrophotometer, Winooski, VT, USA). Caramel (E-150d) was used as a melanoidin standard. Analytical determination was carried out in triplicate. Results were expressed in equivalent milligrams of caramel melanoidins/gram of sample.

#### 2.3.3. Antioxidants

##### Preliminary Information on Antioxidant Composition by Spectral Analysis

UV-Visible spectrum of in liquid IC beverages (4 mg/mL and 10 mg/mL) was obtained using a microplate reader (BioTek Epoch 2 Microplate Spectrophotometer, Winooski, VT, USA). Absorption spectra of samples were recorded from 200 to 700 nm.

##### Phenolic Compounds

For phenolic compounds analysis in liquid IC beverages (4 mg/mL and 10 mg/mL), Folin-Ciocalteu method was adapted to a micro-method format [28]. Folin-Ciocalteu reagent (Sigma-Aldrich, St. Louis, MO, USA) and chlorogenic acid (CGA) (Sigma-Aldrich, St. Louis, MO, USA) standard solution (0.1–0.9 mg/mL) were prepared. Reaction was initiated by adding 10 μL of sample or standard and 150 μL of the Folin solution to a 96-well plate. Blanks from samples and reagent were also analyzed. After 3 min of incubation at 37 °C, 50 μL of sodium carbonate (Sigma-Aldrich, St. Louis, MO, USA) were added. Samples were incubated for 2 h and absorbance was measured at 735 nm. Results were expressed as mg/mL of CGA and measurements were performed in triplicate.

##### Total Anthocyanins

Total anthocyanins content was measured in liquid IC beverages (4 mg/mL and 10 mg/mL) according to Wrolstad and Giusti (2001) [29]. In a 96-well plate, 40 μL of sample and 160 μL of potassium chloride (0.025 M, Sigma-Aldrich, St. Louis, MO, USA) and sodium acetate buffer (0.4 M, Sigma-Aldrich, St. Louis, MO, USA) were added and incubated for 15 min at 37 °C. Absorbance was measured at 520 nm and 700 nm. Cyanidine-3-glucoside (C3G) was used as reference for calibration curve (0.0–0.2 mg/mL) and measurements were performed in triplicate.

##### Overall Antioxidant Capacity

ABTS

ABTS (2,2′-azino-bis(3-ethylbenzothiazoline-6-sulfonic acid)) bleaching method was determined in liquid IC beverages (4 mg/mL and 10 mg/mL) as detailed by Tsao et al. (2003) and rectified by Oki et al. (2006) for its use in a microplate [30,31]. ABTS^•+^ stock solution was prepared by mixing the ABTS^•+^ radical and potassium persulfate (Sigma-Aldrich, St. Louis, MO, USA). Solution was then left to stand for 16h at room temperature. Afterwards, ABTS^•+^ working solution was prepared by diluting the stock solution 1:75 (*v*/*v*) in 5 mM of sodium phosphate buffer at pH 7.4 and adjusted to an absorbance of 0.7 ± 0.02 at 734 nm. Chlorogenic acid (CGA) calibration curve (25–200 μM) was used for antioxidant capacity analysis. Measurements were performed in triplicate and results were expressed as mg/mL of CGA.

FRAP

Antioxidant capacity by FRAP (Ferric Reducing Antioxidant Power Assay) was determined in liquid IC beverages (4 mg/mL and 10 mg/mL) as described by Benzie and Strain (1996) and modified by Tsao et al. (2003) for use in a microplate [30,32]. Experiment was initiated with the preparation of TPTZ 10 mM in 40 mM HCl, ferric chloride hexahydrate (FeCl_3_-6H_2_0) 20 mM, and the FRAP reagent (Sigma-Aldrich, St. Louis, MO, USA): a mixture of 0.3 M of acetate buffer, 2.5 mL of TPTZ solution and 2.5 mL of FeCl_3_-6H_2_O solution. Reaction took place by adding 10 μL of sample and 290 μL of FRAP reagent into a 96-well plate for 10 min at 593 nm. Sample and reagent blanks were also measured. CGA was used for the calibration curve (0.025–0.2 mg/mL) and results were expressed as mg/mL of CGA.

##### Contribution of Melanoidins to the Overall Antioxidant Properties of IC

To determine which compounds contribute to the antioxidant properties of the beverages, each sample was ultra-filtrated using Macrosep Advance Centrifugal Devices (Pall Corporation, Ann Arbor, MI, USA) of a molecular cut membrane of 10 kDa. Samples were centrifuged (Hettich Universal 320R Centrifuge, Andreas Hettich GmbH & Co.KG, Tuttlingen, Germany) at 3000 g for 90 min to separate the high molecular weight (HMW) and low molecular weight (LMW) fractions. HMW fractions were washed three times using same centrifugation conditions. Antioxidant capacity of HMW and LMW fractions of IC beverages (4 and 10 mg/mL) and Tabifruit was analyzed by ABTS and FRAP methods (see methods described above).

### 2.4. Shelf Life Study Under Accelerated Storage Conditions of Liquid IC

Beverage stability was evaluated under different light exposure and temperature conditions. In 10 mL glass vials, samples were tested for 72 h under three different conditions: 40 °C and light (Temp + light), room temperature and light (light) and room temperature and no light exposure (darkness) [33]. Light exposure was carried out with artificial light from a fluorescent lamp of 500 lux of illuminance. Prior to the study, pH of each sample was measured using the pH meter MP 230 (Mettler Toledo, Barcelona, Spain) previously calibrated.

Total phenolic content and ABTS were determined in beverages after the shelf life study as indicated in Section 2.3.3.

#### Color

Color measurement was performed with a Reflectance Integrating Sphere SPECORD 210 Plus (Analytik Jena, Jena, Germany). Samples were analyzed in 2 mL plastic vials and results were obtained through the WinAspect Plus program (Jena, Germany), using the CIE color space L* a* b* as numerical values representing luminosity and color parameters. Samples were analyzed in triplicate. Colors were generated from L* a* b* values using colorizer.org as a high precision color generator [34].

### 2.5. Statistical Analysis

All results were expressed as the mean ± standard deviation (SD). Analysis of variance (ANOVA) and Tukey as a post-hoc test were carried out to determine differences between means. Differences were considered to be statistically significant at *p* < 0.05. Pearson’s correlation coefficient was calculated using the Excel Analysis *ToolPak* (Microsoft, Redmond, WA, USA). XL-Stat version 2020.1.3 (Addinsoft, New York, NY, USA) was used to analyze JAR data. Analysis of variance were conducted on IBM SPSS Statistics 24 (IBM, Armonk, NY, USA).

## 3. Results and Discussion

### 3.1. Nutritional Characterization of CA

Table 1 shows protein and amino acid content (free and total) of the raw material used in this investigation, coffee cascara (CA). Protein content found in CA was 9.55%, which is in accordance to that previously described [10,35,36,37,38].

With regard to amino acid content, no free amino acids were detected in CA. Amino acids present in the raw material derived from the proteins that compose it. Of the total amino acids in CA, 32% corresponded to essential amino acids. Asparagine, glycine and glutamic acid presented the highest values for total amino acids in CA. The main amino acids found in CA corresponded to those reported by Elías in 1979 [39] and also recently described by our research group [10]. Tryptophan was not detected in total amino acids, as the acidic conditions needed for quantification resulted in its hydrolysis.

The lipid content and the fatty acid profile of CA are shown in Table 2. Total fat content found in CA was 2%, which is in line to that reported by other authors (2.3–2.5%) [38,40,41]. In this study, palmitic acid (C16:0) was the main fatty acid in CA (36.02 g/100 g), followed by linoleic acid (C18:2n6c, 21.80 g/100 g), α-linoleic acid (C18:3n3, 17.37 g/100 g) and oleic acid (C18:1n9c, 6.72 g/100 g). The polyunsaturated fatty acids (PUFA) and saturated fatty acids (SFA) ratio was 0.85, i.e., higher than 0.45, which is considered as healthy by the World Health Organization [42].

The main sugars found in CA were glucose (13.45 g/100 g) and fructose (21.70 g/100 g). Mannose (0.06 g/100 g) was also detected in CA. Xylose and sucrose were not detected in this by-product. Values for simple sugars are in line with that reported by Urbaneja et al. (1996) [43].

With regard to fiber content in CA, this by-product presented 47.44% of total dietary fiber (TDF). This dietary fiber is composed of fibers of different nature, being 31.32% insoluble (IDF) and 16.12% soluble (SDF). These values are in line with those reported for CA in other studies and by companies that are using this by-product as a food ingredient [10,44].

Considering micronutrients present in CA, potassium (2284 mg/100 g), magnesium (20.84 mg/100 g), sodium (266.58 mg/100 g) and calcium (54.78 mg/100 g) were the cations detected in this by-product. As for anions, chloride (473.21 mg/100 g), nitrate (127.14 mg/100 g), phosphate (602.79 mg/100 g) and sulfate (193.32 mg/100 g) were also found in CA. Micronutrient values reported in this study are slightly higher than those previously described [39]. In addition, CA presented 69.70 mg/100 g of ascorbic acid. Content of ascorbic acid reported in this study is much higher than that described by The Coffee Cherry Co. for their cascara flour [44]. This difference may be due to many factors influencing the nutritional composition of CA, such as origin, cultivation methods and variety of the coffee plant, among others [45].

### 3.2. Characterization of IC

#### 3.2.1. Nutritional Profile

After an aqueous extraction and freeze-drying process, a powdered extract (IC) was obtained from CA. A nutritional characterization was also carried out in the novel instant beverage. With regard to macronutrients, 65% of the protein present in CA is recovered in IC (6.25%). To the best of our knowledge, this is the first time that the amino acid profile of an aqueous extract of coffee cascara is described (Table 1). IC presented 25 mg/g of free amino acids, 10.19% of them being essential. The aqueous extraction process resulted in a concentration of free amino acids that were not present in CA. From a nutritional point of view, free amino acids would be more bio-accessible in IC compared to CA. Proline (6.77 mg/g), serine (5.71 mg/g) and alanine (2.27 mg/g) were the major free amino acids found in IC. In addition, free aminobutyric acid (GABA) was also detected in IC (0.3 mg/g). GABA is known for its health-promoting properties, such as an anti-hypotensive effect [46]. Together with other fruits, such as melon and apricot, IC could contribute to lower blood pressure. Considering results obtained in free and total amino acids, IC might be a potential sustainable source of these molecules. A recent clinical trial has observed that continuous intake of the amino acid supplements significantly increase muscle amount and improve skin texture in young adult women [47].

With regard to total amino acids, asparagine was the main amino acid in IC (7.79 mg/g), followed by proline (4.82 mg/g) and alanine (2.44 mg/g). Although the claimed health-promoting properties of amino acids are not yet established in terms of a cause–effect relationship after the evaluation of the EFSA Panel, the potential claimed health-promoting properties related to amino acids are growth or maintenance of muscle mass, maintenance of normal muscle function, faster recovery of muscle function/strength/glycogen stores after exercise, faster recovery from muscle fatigue after exercise and skeletal muscle tissue repair [48].

Total fat percentage is significantly higher (*p <* 0.05) in CA (2%) than in IC (0.58%). According to the statement by the European Commission Regulation No 1924/2006, IC would be “low in fat” since the product contains no more than 1.5 g of fat per 100 mL [49]. This instant beverage would even be close to the “fat-free” nutrition claim, which is attributed to products that have no more than 0.5 g of fat per 100 mL. Fatty acid composition of IC mainly includes palmitic acid (C16:0), followed by linoleic acid (C18:2n6) and α-linoleic acid (C18:3n3), similar to that found in CA (Table 2).

The main simple sugars found in IC were fructose (16.19 g/100 g) and glucose (6.02 g/100 g), being lower in IC compared to CA. Xylose (6.02 g/100 g), sucrose (0.08 g/100 g) and mannose (0.03 g/100g) were also detected in IC.

Dietary fiber present in IC was 18.32%, all soluble dietary fiber. As expected, the aqueous extraction process concentrated the SDF present in CA. There are several health promoting properties attributed to SDF, which include reduction in cholesterol level and blood pressure, prevention of gastrointestinal diseases, protection against onset of several cancers, such as colorectal, prostate and breast cancer, and increased mineral bioavailability, among others [50,51]. This novel powdered beverage can reach the nutrition claim of “high in fiber” since the product contains at least 6 g of fiber per 100 g [49]. The health claims attributed to the “high in fiber” nutrition claim are “fiber increases fecal bulk, contributes to normal bowel function and to an acceleration of intestinal transit” [52].

After aqueous extraction, powdered IC was enriched in micronutrients since values of anions and cations in IC were higher compared to CA. Cations in IC were potassium (6701 mg/100 g), magnesium (121.56 mg/100 g), sodium (354.19 mg/100 g) and calcium (109.88 mg/100 g). As for anions, chloride (618.32 mg/100 g), nitrate (489.48 mg/100 g), phosphate (1314 mg/100 g) and sulfate (533.61 mg/100 g) were detected in IC.

The European Commission (EU) regulation N° 1925/2006, indicates that to establish an ingredient as a source of any micronutrient, it must represent at least 15% of the daily recommendation [53]. Recommended daily allowances for potassium and magnesium are 3600 and 300 mg, respectively. Therefore, IC may be considered a “source of potassium and magnesium” since values of potassium and magnesium present in IC represent 15% of the recommended allowance per 100 g of product. The Official Journal of the European Union (No 432/2012) makes the following statement for foods considered as a source of potassium: “Potassium contributes to the normal functioning of the nervous system, muscles, and the maintenance of normal blood pressure” [52]. On the other hand, a product that is a “source of magnesium” is related to the following health claims: “Magnesium contributes to a reduction of tiredness and fatigue, to electrolyte balance, to normal energy-yielding metabolism, to normal functioning of the nervous system, to normal muscle function, to normal protein synthesis, to normal physiological function, to the maintenance of normal bones and teeth and a role in the process of cell division” [52].

With regard to the content of ascorbic acid, IC presented 438.95 mg/100 g. IC can also be considered as a source of vitamin C, considering that the daily recommendation is 60 mg/day. Thus, the following health claims can be attributed to IC: “Vitamin C contributes to the normal functioning of the immune system during intense physical exercise, normal energy metabolism, normal functioning of the nervous system, normal psychological function, protection of cells against oxidative damage, reduction of fatigue and fatigue, regeneration of the reduced form of vitamin E, improvement of iron absorption, and normal collagen formation for the normal functioning of blood vessels, bones, cartilage, gums, skin, and teeth” [52].

Once powdered IC was characterized, two liquid beverages were prepared at 4 and 10 mg/mL. Results of liquid beverages were compared to the commercial cascara infusion Tabifruit. Table 3 shows the composition in nutrients and antioxidants of the three drinks. All samples showed significant differences (*p* < 0.05) in carbohydrate content. Tabifruit presented the lowest values of total carbohydrates (22 g/100 mL) followed by IC at 4 mg/mL (27 g/100 mL) and IC at 10 mg/mL (47 g/100 mL). This carbohydrate fraction may be composed of the soluble dietary fiber previously mentioned and other polysaccharides. For instance, previous studies showed that coffee cascara contains up to 35% of pectin [54].

Regarding glucose content, the same behavior as for total carbohydrates was observed; Tabifruit showed the lowest values (0.02 g/100 mL) and IC at 10 mg/mL the highest (0.05 g/100 mL). As expected, a dose-response effect in total carbohydrates and glucose content was observed in IC at 4 and 10 mg/mL. For both IC beverages, glucose values remained under those established for the nutrition claim “low in sugar” and could even be classed as “sugar-free”. According to the European Commission Regulation (No. 1047/2012), for a food to be considered “low sugar”, it must contain no more than 2.5 g of sugar per 100 mL; and for it to be declared “sugar-free”, the product must not contain more than 0.5 g of sugar per 100 mL [49]. Therefore, considering free glucose content both IC beverages could make the “low sugar” nutrition claim. For the “sugar-free” claim, further quantification of other predominant simple sugars would be necessary. IC is a healthy alternative compared to other instant powdered soft drinks in the Food Data Central Database of the United States Department of Agriculture (USDA), whose sugar content ranges from 29 to 93 g/100 g per product [55].

#### 3.2.2. Impact of MRP on Safety and Health Promoting Properties of IC

##### Safety

MRP compromise the nutritional value, safety and health promoting properties of IC. Since IC contains amino acids (Table 1) and reducing sugars, which are substrates of the Maillard reaction, acrylamide was analyzed to confirm the food safety of the novel beverages. Acrylamide content was below the detection limit for the three beverages. In contrast, it was found in powdered IC at 223 μg/kg. Acrylamide content found in IC is much lower than the limit stablished by the European Commission for coffee and instant coffee, 450 and 900 μg/kg, respectively [7]. Considering acrylamide content, this novel instant beverage would be a safe alternative to instant coffee. However, since acrylamide compromises the food safety of the beverage, shorter drying periods, different drying techniques or using fresh coffee cascara are measures that must be considered to decrease the amount of this compound in IC.

Since asparagine was the main amino acid in IC and this amino acid is known to be responsible for the development of acrylamide, another potential acrylamide mitigation strategy would be treating IC with L-asparaginase [56]. This enzyme is considered to be useful for acrylamide mitigation and to have negligible effects on the general formation of Maillard products. L-Asparaginase can selectively reduce the level of free L-Asn by hydrolyzing it to L-Asp and ammonia, thus specifically removing one of the essential acrylamide precursors [57]. However, in this particular case the preferred acrylamide mitigation strategy would be non-thermal drying procedures of cascara.

Drying of the raw material is a critical step in the conversion of coffee cascara into a safe food ingredient for human consumption. Novel drying methods will ensure the chemical and microbiological safety of cascara. Reducing moisture under 13% is necessary to avoid fungal growth and the consequent production of mycotoxins [58]. In this context, previous research has confirmed the absence of mycotoxins, such as aflatoxin B1, enniantin B and ochratoxin A in a cascara aqueous extract [59].

Another limitation on the use of a coffee by-product as a food ingredient is connected to its caffeine content. Results published so far suggest that caffeine content in an aqueous cascara extract (1.39%) does not need to be considered a safety concern [59]. The caffeine content of IC at 10 mg/mL in 250 mL would be around 34 mg, which is below the EFSA safety level for daily caffeine consumption of 400 mg for the general population, 3 mg/kg b.w. per day for children and adolescents and 200 mg for lactating women [60]. Therefore, almost no limitations on the use of IC for human consumption need to be considered, since pregnant women would have to drink over 1.47 L of IC beverage to exceed the safety level for the fetus.

##### Antioxidant Properties

Overall Antioxidant Capacity and Identification of Antioxidant Compounds

Liquid IC beverages are also a source of antioxidants (Table 3). In both antioxidant capacity determinations (ABTS and FRAP), IC at 10 mg/mL presented a significantly higher (*p* < 0.05) antioxidant capacity (27.58 mg eq. CGA/mL for ABTS and 0.43 mg eq. CGA/mL for FRAP) compared to the other two beverages. Results obtained for the antioxidant capacity corresponded to the total phenolic content (TPC) found in samples, IC being at 10 mg/mL the beverage presenting the highest values for TPC (0.89 mg eq. CGA/100 mL). Pearson’s correlation coefficient was calculated to check for the existence of linear relationships between TPC, ABTS and FRAP of the beverages. A very strong (r = 0.97) or strong (r = 0.79) positive association was found between TPC and ABTS and FRAP, respectively. TPC, but not anthocyanins that were not detected in samples, seem to contribute to the overall antioxidant capacity of IC. Previous studies have reported that coffee cascara is a source of phenolic compounds, such as CGA and protocatechuic acid, which represent more than 80% of the polyphenols analyzed by Heeger et al. (2017) [40].

The presence of anthocyanins in coffee cascara has been previously reported [14,61,62]. However, anthocyanins were not detected in any of the samples using the pH-dependent colorimetric assay (Table 3) and therefore results seem to indicate that they are not the main responsible compounds for the antioxidant properties of IC. A comparative study of fresh and sun-dried grapes indicated a total loss of flavonoids, and in less quantity, of hydroxycinnamic acids (estimated at 62% for sun-dried grapes). This study concluded that the most labile polyphenols were procyanidins and flavan-3-ols, since they were completely degraded in all sun-dried raisin samples [63]. Therefore, anthocyanins might have been degraded during the 21 days of sun drying of the coffee cascara used in the present study, which, in fact, is the most cheap and common method to dry it. However, quantification analysis, such as NMR [64] and HPLC [65], are needed to confirm the absence of anthocyanins in the studied sample. In addition, the three beverages showed a similar UV-Visible absorption spectrum (Figure S1A). A maximum absorption was detected at 280 nm, indicative of the presence of caffeine, proteins and phenolic compounds, and at 325 nm, which would correspond to CGA and caffeic acid [66]. This analysis would also support the absence of anthocyanins that absorb at 520 nm [29] (Figure S1B), since none of the beverages seemed to absorb at this wavelength (Figure S1A).

With regard to Maillard reaction products, melanoidins were detected in the three beverages at 1.48 mg eq. caramel/mL, 0.54 mg eq. caramel/mL, 1.2 mg eq. caramel/mL, for IC (10 mg/mL), IC (4 mg/mL and Tabifruit, respectively. To the best of our knowledge, this is the first time the presence of melanoidins and the occurrence of the Maillard reaction are described in coffee cascara. Melanoidins may have been formed during sun drying of the raw material for 21 days, as occurs during the dehydration of figs, dates and raisins under similar conditions to those employed in cascara processing [11]. In addition, beverages absorbed over the full wavelength range of 200–700 nm, which is characteristic of melanoidins [5]. CGA and melanoidins, usually have a maximum absorption close to 360 nm, which might indicate the linkage of CGA molecules to the structure of melanoidins by non-covalent interactions [5,67,68].

Contribution of MRP to the Antioxidant Properties of IC

In order to identify which compounds were responsible for the antioxidant capacity of IC, the three beverages were ultra-filtrated by a 10 kDa cut-off membrane. Table 4 shows the overall antioxidant capacity of the high (HMW) and low (LMW) molecular weight fractions of the three beverages measured by ABTS and FRAP. Antioxidant capacity analyzed by ABTS and FRAP of the LMW fraction of the three beverages did not differ significantly (*p* > 0.05) among samples. The HMW fractions of the three beverages was significantly (*p* < 0.05) more antioxidant compared to the LMW fraction. The HMW fraction of IC at 10 mg/mL presented the highest values of antioxidant capacity analyzed by ABTS (82.85 mq eq. CGA/mL) and FRAP 1.08 mg eq. CGA/mL). For both antioxidant capacity determinations, the HMW fraction of Tabifruit presented the lowest values. Results seem to indicate that HMW compounds (>10 kDa), such as melanoidins seem to be the main contributors of the overall antioxidant capacity of IC.

Melanoidins are high molecular weight, brown-colored and nitrogen-containing compounds generated in the late stages of the Maillard reaction [4]. Rufián-Henares and Pastoriza stated that melanoidins are great contributors to the overall antioxidant intake in the Spanish diet. In addition, these melanoidins come mainly from coffee, followed by biscuits, beer and chocolate [3,69]. Many health-promoting properties are attributed to melanoidins, such as antioxidant, antimicrobial, anti-inflammatory, antihypertensive or prebiotic activity [4]. Melanoidins have also been isolated from other coffee by-products, such as silverskin, and have shown antioxidant properties in vitro and a dietary fiber effect in vivo [67]. Melanoidins extracted from spent coffee grounds, the last coffee by-product generated during the beverage elaboration, have also shown antioxidant capacity in vitro [70]. IC may be another sustainable source of melanoidins that would contribute to the antioxidant intake of the global population.

### 3.3. Shelf Life Study Under Accelerated Storage Conditions of Liquid IC

**Figure 1:**
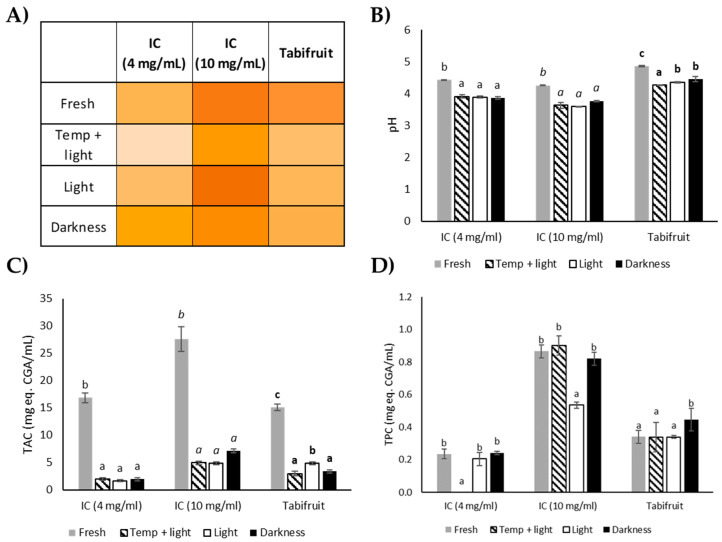
Photosensitivity and heat resistance of IC at 4 mg/mL, IC at 10 mg/mL and Tabifruit. (**A**) Color differences (colorizer.org as a tool for color generation [34]); (**B**) pH; (**C**) Total antioxidant capacity (TAC) determined by ABTS; (**D**) Total phenolic content (TPC). Bars represent the mean values and the error bars denote the standard deviation. Different letters on bars indicate significant differences in each beverage sample (Tukey Test, *p* < 0.05).

For the stability study, the three liquid beverages were exposed to different conditions of light and temperature for 72 h. Figure 1 shows the differences in color, pH, total antioxidant capacity (TAC) and total phenolic content (TPC) in each beverage exposed to different conditions of light and temperature. As for color, significant differences (*p <* 0.05) were found in L a* b* parameters when the three beverages were exposed to 40 °C and light for 72 h (Table S1). Color alteration after temperature al light exposure can be easily visualized in Figure 1A. Tabifruit was the only sample that presented a significant change (*p <* 0.05) to light treatment, which might suggest that CA has a higher susceptibility to light exposure (Table S1). In general, there is a significant decrease (*p* < 0.05) in the red tonality (a*) of all beverages after temperature and light exposure (Table S1).

For IC at 4 and 10 mg/mL, parameters L and a* correspond linearly to IC concentration (Table S1). Torres et al. (2019) studied the color parameters in coffee cascara before and after drying process. They observed a darker coloration in parameter a* (red color) in the dry samples, concluding that it may be related to the browning processes as effects of the temperature used in the drying of the cascara [71]. Altogether, melanoidins generated during the drying process of coffee cascara seem to be responsible for the color of IC and Tabifruit. This is in accordance to the UV-Visible spectra of the beverages (Figure S1A), which absorb throughout the whole spectrum in a similar way to the caramel standard used to determine melanoidin content (Figure S1B) [72].

Regarding pH results, pH values of all beverages significantly decreased (*p* < 0.05) after the stability study compared to the fresh preparation of the beverages (Figure 1B). These results seem to indicate that temperature and light are determining factors for changes in pH. Nicoli et al. explain that a drop in pH may occur as a consequence of reactions that might be related to non-enzymatic browning, the Maillard reaction, between carbohydrates and amino acids that occurs also during storage [73,74]. The pH is a determining factor for flavor, color, and shelf life of flavored beverages, and it is estimated that the optimal pH to prevent the growth of bacteria and accentuate flavor notes is between 3 and 4 [75]. Other authors state that drinks with acidic pH show good color stability under refrigerated storage conditions to maintain their phenolic content at 90% for a period of 4 months approximately [76]. Therefore, the low pH of IC is suitable for the preservation of the bioactive compounds present in the beverage.

Figure 1C shows results for TAC of the beverages after the stability study. A significant decrease (*p* < 0.05) in antioxidant capacity was observed in all samples compared to the fresh preparation. Considering the TPC (Figure 1D), temperature and light and only light exposure produced a significant decrease in TPC in IC at 4 and 10 mg/mL, respectively. Keeping the liquid preparation in darkness seemed to be the condition that best preserved the phenolic compounds present in IC and Tabifruit.

Considering the composition in nutrients and antioxidants, together with the stability analysis, powdered IC could be considered as the best option for its future commercialization. The process of obtaining IC is green, simple and could be easily carried out using the same facilities used to produce instant coffee. A stability study can predict and optimize the most suitable package conditions to conserve the beverage [33]. Taking into account results from the stability study, IC could be packaged as an instant beverage format to dissolve in either warm or cold water at 10 mg/mL, a similar dosage as the one used for commercially available instant coffee products. Powdered IC would have longer shelf life and less distribution costs, but less expensive concentration and drying techniques, such as vacuum drying at low temperatures (50 °C) and for short periods [77], are needed to implement this procedure in coffee producing countries.

## 4. Conclusions

A safe instant beverage with antioxidant properties has been obtained to which the following nutrition claims can be assigned: “low fat”, “low sugar”, “high in fiber” and “source of potassium, magnesium and vitamin C”. For the first time, cascara beverage color was attributed to the presence of melanoidins. The shelf life study seemed to indicate that IC beverages in solution are more susceptible to color changes by light and temperature (40 °C) exposure. A package is therefore suggested that protects the product from light and is stored in a cool, dry place. Although very low levels of acrylamide were reported, melanoidins with potential health-promoting properties have also been formed. These melanoidins’ high molecular weight compounds (>10 kDa) seem to contribute mostly to the overall antioxidant capacity of IC. The novel powdered instant beverage developed in the present study, IC, is a potential sustainable alternative for instant coffee, with low caffeine and acrylamide levels and a healthy composition in nutrients and antioxidants that would allow the whole recovery of the by-product in two novel ingredients (IC and a dietary fiber fraction).

## Figures and Tables

**Table 1:** Total protein (%) and free and total amino acid content (mg/g) of raw dry coffee cascara (CA) and powdered Instant Cascara beverage (IC).

	CA		IC	
Total Protein (%)	9.55 ± 0.11 ^b^		6.25 ± 0.27 ^a^	
Amino Acids (mg/g)	Free	Total	Free	Total
Aminobutyric acid (GABA)	N.D.	N.A.	0.3 ± 0.00	N.A.
Glutamic acid (Glu)	N.D.	2.13 ± 0.04 ^a^	0.40 ± 0.01	2.11 ± 0.17 ^a^
Alanine (Ala)	N.D.	2.06 ± 0.11 ^a^	2.27 ± 0.01	2.44 ± 0.05 ^a^
Arginine (Arg)	N.D.	0.48 ± 0.03 ^a^	1.39 ± 0.02	1.05 ± 0.04 ^b^
Asparagine (Asp)	N.D.	2.84 ± 0.14 ^a^	1.91 ± 0.05	7.79 ± 0.37 ^b^
Cysteine (Cis)	N.D.	0.18 ± 0.00 ^a^	0.17 ± 0.01	0.22 ± 0.02 ^a^
Phenylalanine (Phe)	N.D.	0.98 ± 0.10 ^a^	0.17 ± 0.01	0.31 ± 0.01 ^a^
Glycine (Gly)	N.D.	2.71 ± 0.17 ^a^	0.30 ± 0.27	1.19 ± 0.04 ^a^
Histidine (His)	N.D.	0.52 ± 0.10 ^b^	0.08 ± 0.01	0.16 ± 0.01 ^a^
Isoleucine (Ile)	N.D.	0.63 ± 0.05 ^b^	0.05 ± 0.00	0.18 ± 0.04 ^a^
Leucine (Leu)	N.D.	1.17 ± 0.03 ^b^	0.05 ± 0.01	0.27 ± 0.04 ^a^
Lysine (Lys)	N.D.	0.38 ± 0.01 ^a^	0.03 ± 0.01	0.21 ± 0.01 ^a^
Methionine (Met)	N.D.	0.25 ± 0.02 ^a^	N.D.	0.07 ± 0.04 ^a^
Proline (Pro)	N.D.	1.60 ± 0.01 ^a^	6.77 ± 0.02	4.82 ± 0.12 ^b^
Serine (Ser)	N.D.	1.91 ± 0.02 ^a^	5.71 ± 0.01	2.08 ± 0.05 ^a^
Tyrosine (Tyr)	N.D.	0.63 ± 0.05 ^a^	N.D.	0.68 ± 0.01 ^a^
Tryptophan (Trp)	N.D.	N.A.	N.D.	N.A.
Threonine (Thr)	N.D.	0.96 ± 0.03 ^b^	0.05 ± 0.01	0.35 ± 0.02 ^a^
Valine (Val)	N.D.	1.27 ± 0.02 ^a^	0.30 ± 0.01	0.60 ± 0.07 ^a^
Totals (mg/g)	N.D.	25.05 ± 0.16 ^a^	20.43 ± 0.37	29.78 ± 1.97 ^b^
EAA (% total)	N.D.	32.07 ± 0.07 ^b^	10.19 ± 0.01	13.04 ± 0.25 ^a^
BCAA (Val + Leu + Ile) (% total)	N.D.	14.83 ± 0.10 ^b^	1.92 ± 0.01	4.28 ± 0.14 ^a^
AAA (Phe + Tyr + Trp) (% total)	N.D.	7.77 ± 0.15 ^b^	0.8 ± 0.01	4.03 ± 0.00 ^a^

EAA, essential amino acids; BCAA, branched-chain amino acids; AAA, aromatic amino acids. N.D., not detected; N.A., not analyzed. Results are expressed as mean ± SD. Different superscript letters indicate significant differences for total protein and total amino acids (Student’s T, *p* < 0.05).

**Table 2:** Total fat content (%) and fatty acid profile (g/100g) of raw dry coffee cascara (CA) and powdered Instant Cascara beverage (IC).

	CA	IC
Total Fat (%)	2.00 ± 0.50 ^b^	0.58 ± 0.18 ^a^
Fatty Acid Profile (g/100 g)		
C12:0	0.10 ± 0.02 ^a^	N.D ^a^
C14:0	1.18 ± 0.02 ^a^	5.77 ± 0.41 ^b^
C15:0	0.37 ± 0.01 ^a^	4.02 ± 0.59 ^a^
C16:0	36.02 ± 0.32 ^a^	30.54 ± 3.27 ^b^
C16:1n7	3.04 ± 0.19 ^a^	3.90 ± 0.23 ^a^
C17:0	0.56 ± 0.01 ^a^	N.D ^b^
C18:0	5.64 ± 0.22 ^a^	4.54 ± 0.37 ^a^
C18:1n7c	1.79 ± 0.05 ^a^	N.D ^b^
C18:1n9c	6.72 ± 0.37 ^a^	10.82 ± 1.80 ^a^
C18:2n6c	21.80 ± 0.34 ^a^	15.83 ± 1.00 ^a^
C18:3n3	17.37 ± 0.24 ^a^	14.84 ± 1.46 ^a^
C20:0	2.82 ± 0.07 ^a^	N.D ^a^
C20:1n9	0.09 ± 0.00 ^a^	N.D ^a^
C20:2n6	0.09 ± 0.00 ^a^	N.D ^a^
C20:3n3	0.19 ± 0.04 ^a^	N.D ^a^
C20:5n3	N.D ^a^	N.D ^a^
C21:0/C20:3n6 *	0.10 ± 0.01 ^a^	N.D ^a^
C22:0	0.64 ± 0.04 ^a^	N.D ^b^
C22:6n3	0.76 ± 0.60 ^a^	9.74 ± 5.54 ^a^
C23:0	0.16 ± 0.02 ^a^	N.D ^a^
C24:0/C22:5n3 *	0.57 ± 0.00 ^a^	N.D ^b^
SFA (%)	47.48 ± 0.10 ^a^	44.87 ± 4.65 ^a^
MUFA (%)	11.64 ± 0.12 ^a^	14.72 ± 1.57 ^a^
PUFA (%)	40.88 ± 0.02 ^a^	40.41 ± 3.08 ^a^

SFA, saturated fatty acids; MUFA, monounsaturated fatty acids; PUFA, polyunsaturated fatty acids; N.D., not detected. The values indicate the mean ± SD. Different superscript letters indicate significant differences (Student’s T. *p* < 0.05). * The chromatographic method does not allow the separation of the fatty acids C21:0 and C20:3n6; and C24:0 and C22:5n3, so the value obtained may be due to either one or the sum of both.

**Table 3:** Nutrients (total carbohydrates (g/100 mL), glucose content (g/100 mL)) and non-nutrient antioxidants of the three beverages IC (4 mg/mL), IC (10 mg/mL) and Tabifruit.

	IC (4 mg/mL)	IC (10 mg/mL)	Tabifruit
Nutrients			
Total carbohydrates (g/100 mL)	27.50 ± 0.07 ^b^	47.48 ± 0.20 ^c^	22.22 ± 0.06 ^a^
Glucose (g/100 mL)	0.04 ± 0.01 ^b^	0.05 ± 0.01 ^c^	0.02 ± 0.01 ^a^
Antioxidants			
Total phenolic content (mg eq. CGA/mL)	0.25 ± 0.0 ^a^	0.89 ± 0.0 ^c^	0.37 ± 0.0 ^b^
Anthocyanins	N.D.	N.D.	N.D.
ABTS (mg eq. CGA/mL)	16.82 ± 0.9 ^b^	27.58 ± 2.3 ^c^	15.05 ± 0.6 ^a^
FRAP (mg eq. CGA/mL)	0.15 ± 0.0 ^a^	0.43 ± 0.0 ^c^	0.34 ± 0.0 ^b^

CGA, chlorogenic acid. Each value represents the mean ± SD. Different letters indicate significant differences *(p* < 0.05) between samples in the same row. Different superscript letters indicate significant differences between samples (Tukey Test, *p* < 0.05).

**Table 4:** Antioxidant capacity analyzed by ABTS (mg eq. CGA/mL) and FRAP (mg eq. CGA/mL) of the high molecular weight (HMW, >10 kDa) and low molecular weight (LMW, <10 kDa) fractions of beverages IC (4 mg/mL), IC (10 mg/mL) and Tabifruit separated by ultrafiltration.

	IC (4 mg/mL)		IC (10 mg/mL)		Tabifruit	
	HMW	LMW	HMW	LMW	HMW	LMW
ABTS	61.25 ± 0.49 ^b^	0.92 ± 0.01 ^a^	82.85 ± 0.19 ^d^	0.92 ± 0.00 ^a^	66.68 ± 0.48 ^c^	0.93 ± 0.00 ^a^
FRAP	0.61 ± 0.08 ^b^	0.12 ± 0.00 ^a^	1.08 ± 0.02 ^d^	0.25 ± 0.01 ^a^	0.90 ± 0.09 ^c^	0.14 ± 0.00 ^a^

Values indicate the mean ± standard deviation and different superscript letters denote significant differences between each row (Tukey Test. *p* < 0.05).

## Supplementary Materials

The following are available online at https://www.mdpi.com/2304-8158/9/5/620/s1, Figure S1: (A) UV-Visible absorption spectrum of the three beverages, measured in a wavelength range of 200 to 700 nm. (B) UV-Visible spectrum of caramel E-150d (Car) and cyanidine-3-glucoside (C-3) at pH 1 and 4.5; Table S1: Measurement of color (L, a*, b*) of the three beverages IC (4 mg/mL), IC (10 mg/mL) and Tabifruit after stability test for 72 h. Studied conditions were: Fresh, 40 °C and light (Temp + light), room temperature and light (light) and room temperature and no light exposure (darkness).
