# Supplementary material for: Assessment of Healthy and Harmful Maillard Reaction Products in a Novel Coffee Cascara Beverage: Melanoidins and Acrylamide
Source: Foods. 2020 May 12;9(5):620. doi: 10.3390/foods9050620 (PMC7278827; doi:10.3390/foods9050620)
Supplement: Supplementary file 1 [file foods-09-00620-s001.pdf]

foods

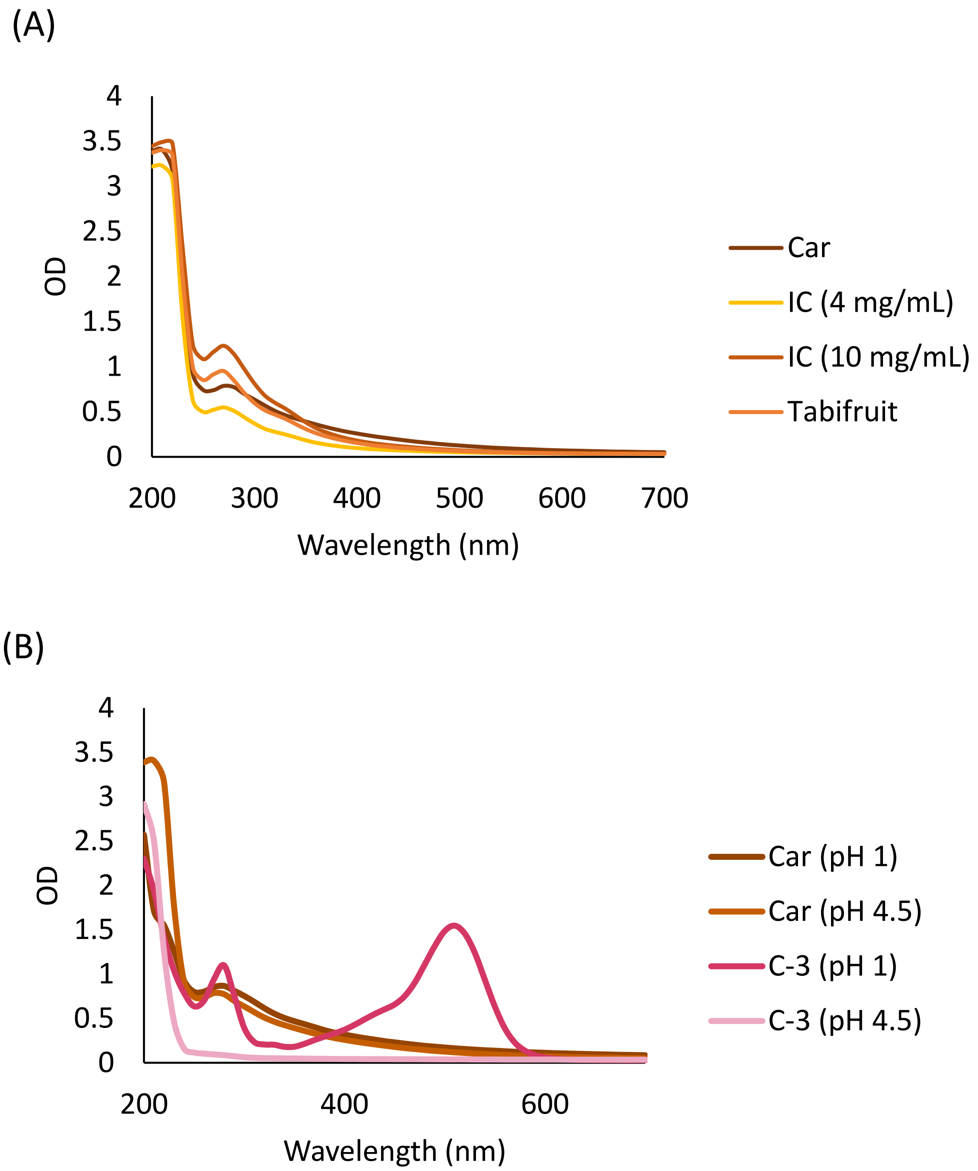

Figure S1. (**A**) UV-Visible absorption spectrum of the three beverages and caramel E-150d (Car) at 1 mg/mL, measured in a wavelength range of 200 to 700 nm. (**B**) UV-Visible spectrum of caramel E-150d (Car) at 1 mg/mL and cyanidine-3-glucoside (C-3) at 0.15 mg/mL at pH 1 and 4.5.

Table S1. Measurement of color (L, a*, b*) of the three beverages IC (4 mg/mL), IC (10 mg/mL) and Tabifruit after stability test for 72 h. Studied conditions were: Fresh, 40 ºC and light (Temp + light), room temperature and light (light) and room temperature and no light exposure (darkness).

		**Color**		
		L	a*	b*
**IC (4 mg/mL)**	Fresh	78.45 ± 0.2^{b}	17.94 ± 0.4^{c}	56.50 ± 0.7^{c}
	Temp + light	88.47 ± 0.5^{c}	4.52 ± 0.2^{a}	20.72 ± 0.3^{a}
	Light	79.89 ± 1.3^{b}	13.66 ± 1.2^{b}	48.74 ± 0.4^{b}
	Darkness	74.62 ± 0.8 ^{a}	24.27 ± 0.6 ^{d}	73.76 ± 0.7^{d}
**IC (10 mg/mL)**	Fresh	60.83 ± 0.8^{a}	32.53 ± 0.5^{c}	59.99 ± 4.1^{a}
	Temp + light	69.59 ± 2.00^{b}	20.78 ± 3.3^{a}	67.40 ± 0.6^{b}
	Light	57.55 ± 2.9^{a}	34.97 ± 1.7^{c}	83,70 ± 2.7^{d}
	Darkness	65.77 ± 2.4^{b}	26.62 ± 0.9^{b}	73.93 ± 0.9^{c}
**Tabifruit**	Fresh	67.74 ± 2.2^{a}	27.49 ± 1.3^{c}	57.75 ± 7.4^{b}
	Temp + light	80.71 ± 1.2^{b}	11.89 ± 0.6^{a}	47.36 ± 0.9^{a}
	Light	78.84 ± 1.4^{b}	14.72 ± 0.5^{b}	53.63 ± 1.2^{ab}
	Darkness	76.21 ± 2.2^{b}	15.60 ± 0.9^{b}	57.61 ± 1.3^{b}

Values indicate the mean ± standard deviation. Different letters represent significant differences for each sample and parameter (Tukey Test. $p < 0.05$).
